# Innovative application of green surfactants as eco-friendly scale inhibitors in industrial water systems

**DOI:** 10.1038/s41598-024-78879-1

**Published:** 2024-11-14

**Authors:** E. Khamis, D. E. Abd-El-Khalek, Manal Fawzy, Kamal A. Soliman, A. M. Abdel-Gaber, J. M. Anwar

**Affiliations:** 1 Chemistry Department, Faculty of Science, Alexandria University, Alexandria, 21321 Egypt; 2 Science and Innovation Center of Excellence, SICE, Egyptian Russian University, Badr, Egypt; 3 National Institute of Oceanography and Fisheries (NIOF), Cairo, Egypt; 4 Environmental Sciences Department, Faculty of Science, Alexandria University, Alexandria, 21511 Egypt; 5 Green Technology Group, Faculty of Science, Alexandria University, Alexandria, 21511 Egypt; 6 Chemistry Department, Faculty of Science, Benha University, Benha, 13518 Egypt; 7 Water Company, Holding Company of Water & Wastewater, P.O. Box: 21511, Alexandria, Egypt

**Keywords:** Scale inhibition, Rhamnolipids, Casein, DFT, Monte Carlo simulation, Environmental sciences, Chemistry, Materials science

## Abstract

Scale deposition poses significant challenges in various industrial utilities, necessitating the development of eco-friendly scale inhibitors in line with environmental regulations. This study investigates the potential of two natural surfactants, Casein and Rhamnolipid, as innovative inhibitors for calcium carbonate (CaCO_3_) scale formation, offering an alternative to traditional water treatment chemicals. The anti-scaling characteristics of these two green surfactants were performed using conductivity and electrochemical impedance spectroscopy (EIS) techniques. Additionally, X-ray diffraction (XRD) and scanning electron microscopy (SEM) were utilized to analyze the morphology of CaCO_3_ crystals and understand the structural changes induced by surfactant interaction. The results revealed that Rhamnolipids significantly outperform Casein in suppressing scale formation, attributed to the adsorption of their multiple functional groups onto scale microcrystals. This adsorption modifies the crystal structure and inhibits further growth. Computational studies were employed to investigate the inhibition mechanism of these surfactants. The spatial and electrical configurations of optimal molecular structures are also analyzed using the Density Functional Theory (DFT) approach. Monte Carlo simulations on the CaCO_3_ (104) surface demonstrated that Rhamnolipids exhibit superior inhibition compared to Casein, as evidenced by their higher adsorption energy, indicating a more stable binding to the surface. Overall, this research highlights the potential of these natural surfactants as sustainable scale inhibitors, particularly in industries such as food and pharmaceuticals, thereby contributing to environmentally friendly water treatment solutions.

**Supplementary Information:**

The online version contains supplementary material available at 10.1038/s41598-024-78879-1.

## Introduction

Scale deposits in industrial water systems pose significant challenges, leading to both technical complications and substantial financial losses. These deposits obstruct water flow in pipes, reduce the efficiency of desalination processes, and impair heat transfer in heat exchangers^1–7^. Such issues not only increase operational costs but also necessitate frequent maintenance and downtime, impacting overall productivity.

The chemistry of feed water plays a crucial role in scale formation, with various factors contributing to the types of scales produced^7–9^. Common types include alkaline scales, such as calcium carbonate (CaCO_3_), non-alkaline scales like calcium sulfate (CaSO_4_), and silica-based scales. Among these, calcium carbonate is the most prevalent, arising from the presence of bicarbonate and calcium ions found in various water sources, including surface water, groundwater, brine, and industrial effluents^7,10,11^.

Numerous strategies have been developed to prevent and control scale formation in industrial water systems. Traditional methods include acid treatment, additive treatment, and mechanical cleaning, each with varying degrees of effectiveness and environmental impact^12–14^. Among these, chemical inhibitors stand out for their ability to regulate crystal growth and alter the morphology of scale-forming compounds. These inhibitors work by interfering with the nucleation and growth processes of calcium carbonate (CaCO_3_) crystals, thereby mitigating scale deposition within pipes and equipment^15,16^.

Among the widely used commercial scale inhibitors, phosphorus-containing compounds are a significant portion of highly effective agents. However, their discharge into oceans and seas can lead to eutrophication, a process that promotes rapid algal growth, covers water surfaces, and ultimately degrades water quality, posing serious threats to environmental health^17,18^. Additionally, these compounds can facilitate the deposition of calcium phosphate, further complicating scale management^19^. In response to these environmental concerns and regulatory limitations, research has increasingly focused on “green scale inhibitor chemistry^18,20–23^. In Our previous work, natural extracts and bio-polymers have gained attention as potential scale inhibitors, contributing to the development of environmentally friendly water treatment chemicals^18,22–26^.

Naturally-based surfactants have emerged as a prominent class of eco-friendly surfactants, offering several advantages, including low toxicity, biodegradability, affordability, and ease of production. These surfactant molecules typically consist of a polar hydrophilic “head” and a non-polar hydrophobic “tail.” In aqueous solutions, surfactants can adsorb onto surfaces through chemisorption or physisorption processes. Casein is recognized as a natural surfactant that facilitates the formation and stabilization of emulsions. Although it exhibits relatively low surface activity, Casein can function effectively as a high-mass surfactant^27^. In contrast, microbial surfactants, also known as biosurfactants, are produced by various microorganisms and have a wide range of applications across industries such as food processing, pharmaceuticals, petrochemicals, environmental management, and cosmetics. One notable class of biosurfactants is Rhamnolipids, which are synthesized by Pseudomonas species^28^. Rhamnolipids are glycolipids composed of one or two rhamnose molecules linked to one or two β-hydroxydecanoic acid molecules^29^.

**Fig. 1:**
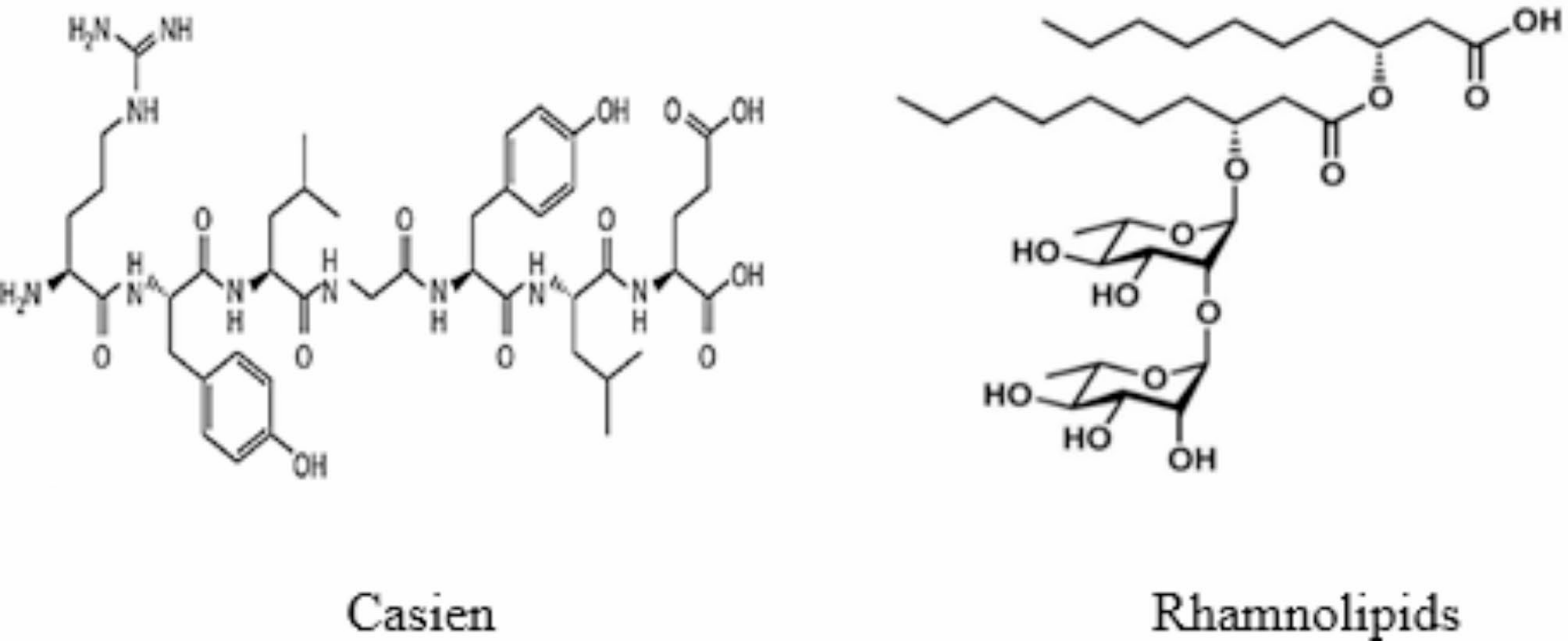
Structural formula of Casien and Rhamnolipids.

Given the biocompatibility, biodegradability, and structural diversity (Fig. 1) of Casein and Rhamnolipids^30–33^, this study aims to explore their potential as eco-friendly alternatives to traditional chemical inhibitors in industrial water systems. This shift toward sustainable solutions underscores the importance of developing innovative approaches to scale management that prioritize environmental safety. The efficiency of these surfactants as inhibitors for CaCO_3_ scale precipitation was investigated using static, electrochemical, and morphological examinations. Additionally, Density Functional Theory (DFT) and Monte Carlo simulations were employed to analyze the inhibitory mechanisms of Rhamnolipids and Casein. The primary objective is to identify the active sites that govern the adsorption of these surfactants onto the CaCO3 (104) surface. By integrating theoretical approaches, we aim to elucidate the electronic properties and adsorption dynamics, focusing on the specific molecular centers of Rhamnolipids and Casein that are crucial for their interactions with the CaCO_3_ surface.

## Results and discussion

### Assessment of scale inhibition effectiveness of rhamnolipids and casein

#### Conductivity measurements

**Fig. 2:**
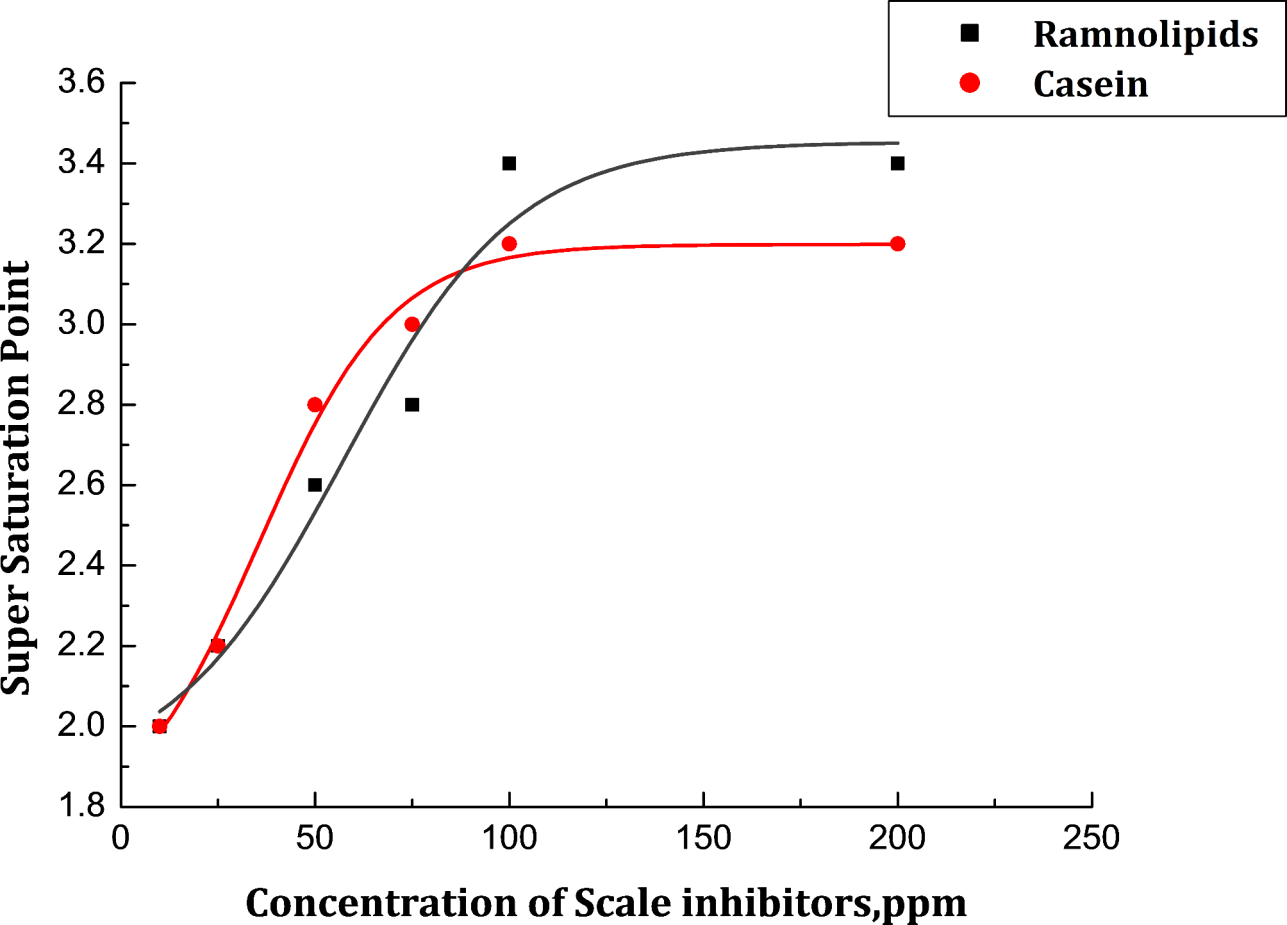
Variation of supersaturation point of CaCO_3_ in presence of various concentrations of Rhamnolipids (**a**) and Casein (**b**).

The effectiveness of the suggested inhibitors in preventing CaCO_3_ scale precipitation was initially assessed using an electrical conductivity test. In this section, sodium carbonate was added to calcium chloride solution to precipitate calcium carbonate while observing the electrical conductivity (see Supplementary Fig. S1). The conductivity test can be considered as a screening procedure used to evaluate the ability of an inhibitor to impede or prevent the development of critical CaCO_3_ nuclei in supersaturated solution. Nucleation is recognized as the initial stage of precipitation. The important point in homogeneous nucleation is the supersaturation point or S. The homogenous nucleation rate increases when the ion product reaches this particular point, causing CaCO_3_ precipitation (scaling)^34,35^. Figure 2 shows the variation of the calcium carbonate scale’s supersaturation point with Casein and Rhamnolipid concentrations. It can be seen that the addition of Rhamnolipids and Casein delays the point of supersaturation, which in turn prevents calcium carbonate precipitation. The overall results indicated that Rhamnolipids is more efficient than Casein in terms of antiscaling performance, however, Casein has a variety of functional groups. In casein, the strong coordination between (-CO-NH) and (COOH) groups and the calcium cations may be the reason for the poor scale inhibition. As a result of this interaction, less scale inhibitor is adsorbed on the microcrystals and more inhibitor is required to achieve the required surface coverage to cause the inhibition^36^.

#### Electrochemical measurements

**Fig. 3:**
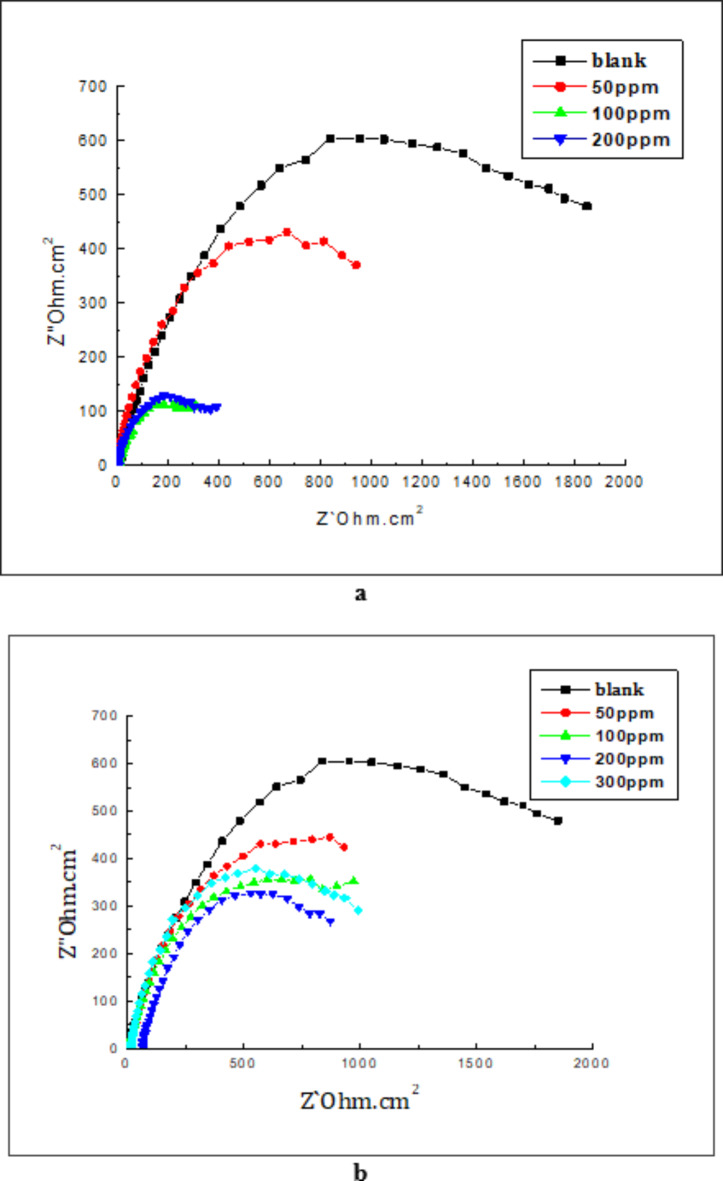
Electrochemical impedance spectra of steel in scaling solution without and with different Rhamnolipids (**a**) and Casein (**b**) concentrations.

This test accelerates the deposition of CaCO_3_ scales on the steel surface electrochemically and examines the ability of a substance to inhibit the formation of these scales. Impedance spectra for the steel following CaCO_3_ precipitation using cathodic polarization in scaling solution both in the absence and in the presence of different Rhamnolipid and Casein concentrations are shown in Fig. 3. Nyquist diagrams of the two surfactants display a characteristic of depressed semi-circles. As can be observed, the presence of surfactant causes a decline in the size of the deformed semicircles because a tiny scale’s insulation layer is formed which reduces the charge transfer resistance^35^. The equivalent circuits shown in Supplementary Fig. S2 online were employed for the analysis of the experimental data from the impedance plots^35^ The fitting of the impedance curve was presented in Fig. S3.

Results of the impedance spectrum’s computer fitting obtained for the cathodically polarized steel electrode in scaling solution containing both surfactants after three hours are displayed in Table 1. According to the results, the charge transfer resistance R_ct_, which is inversely proportional to the quantity of scale formed, decreased as Casein and Rhamnolipid concentrations increased. This behavior reflects these substances’ ability to inhibit scale formation^35^.

**Table 1:** The computer-fitted results of impedance spectrum of the steel electrode, which was polarized cathodically for three hours in a CaCl_2_ brine solution with varying amounts of Casein and Rhamnolipids.

Scale inhibitor	Conc.	*R*_s_	Q	*n*	*R*_ct_	% Inhibition
	ppm	ohm.cm^2^	µF		Ohm.cm^2^	
Blank	0	9.15 ± 0.074	149.9 ± 0.836	0.675	2207 ± 25.1	**–**
Rhamnolipid	50	7.859 ± 0.052	337.7 ± 1.94	0.78	1430 ± 18.6	35.20%
	100	4.46 ± 0.195	51 ± 2.28	0.602	419 ± 7.3	81%
	200	5.01 ± 0.048	27 ± 3.36	0.729	427 ± 4.9	80.60%
Casein	50	5.91 ± 0.038	613 ± 7.63	0.706	1464 ± 29.2	33.60%
	100	6.12 ± 0.042	400 ± 5.38	0.724	1140 ± 16.0	48.30%
	200	63.47 ± 0.68	327.6 ± 7.47	0.76	971.6 ± 16.9	56%
	300	9.96 ± 0.062	276 ± 4.18	0.767	1130 ± 14.5	48.80%

**Fig. 4:**
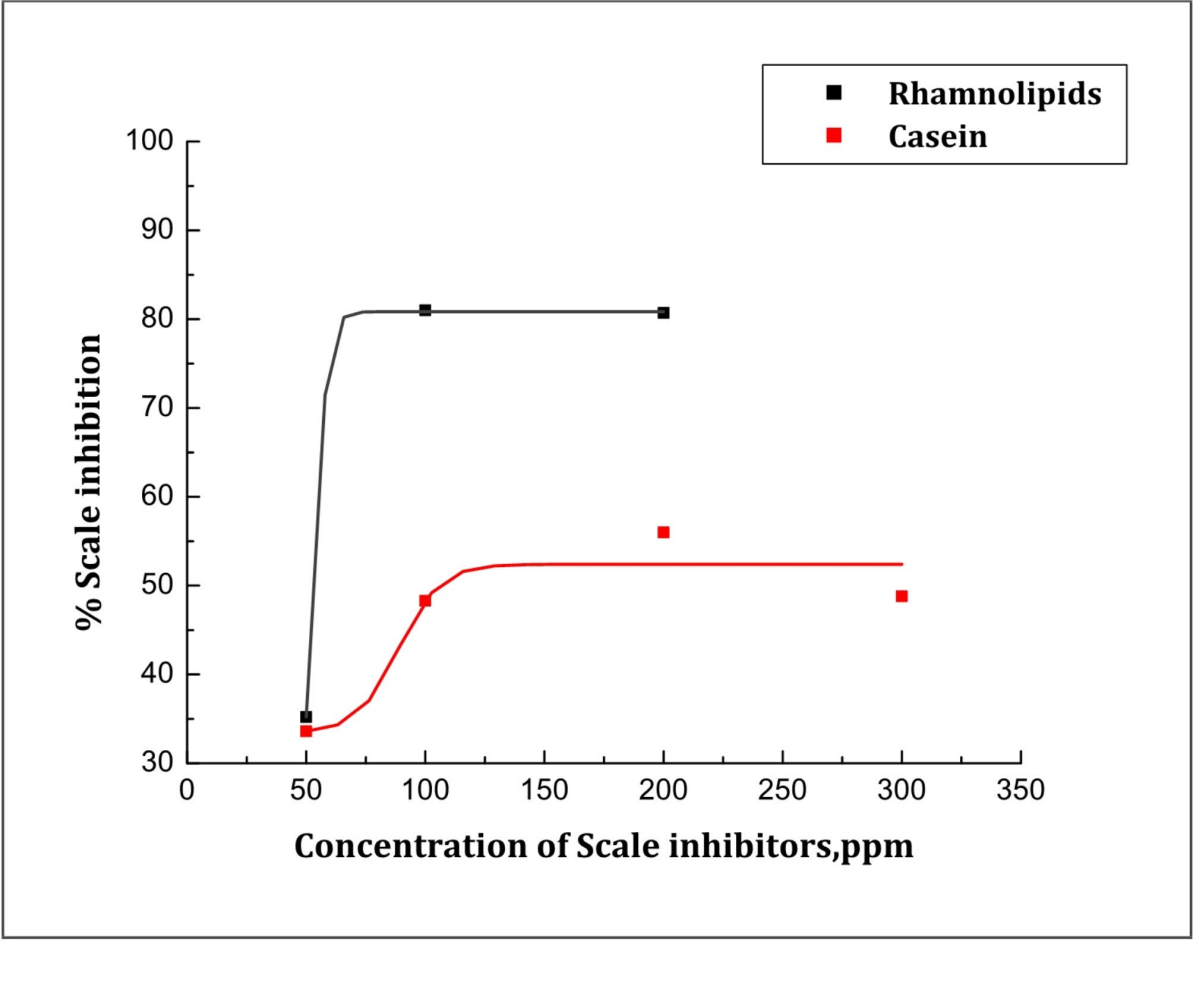
Variation of % scale inhibition of calcium carbonate scales with Rhamnolipids (**a**) and Casein (**b**) concentrations.

The following equation can be utilized to calculate the percentage of scale inhibition^35^:

1 $$\% {\text{ Scale Inhibition }}={\text{ }}[{\left( {{\text{Rct}}} \right)_{\text{o}}} - {\left( {{\text{Rct}}} \right)_{\text{i}}}]{\text{ }}{\left( {{\text{Rct}}} \right)_{\text{o}}} \times {\text{1}}00$$

where (Rct)o and (Rct)i are the charge transfer resistances of the steel electrode following three hours of cathodic polarization at − 0.95 V (vs. SCE) in the scale environment without and with tested inhibitors, respectively. Figure 4 illustrates that when Rhamnolipids and Casein are present at the ideal concentrations, the maximal CaCO_3_ scale inhibition is 81% and 56%, respectively. This suggests that the inhibitor caused the carbonate crystals to become distorted and prevented them from adhering to the steel surface.

#### Morphological examination of CaCO3 SCALES

##### Scanning electron microscopy (SEM)

**Fig. 5:**
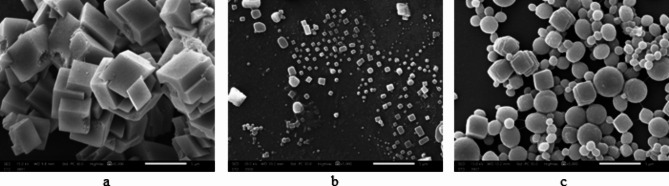
SEM photos of precipitated CaCO_3_ crystals in the absence (**a**) and presence of Rhamnolipids (**b**) and Casein (**c**).

SEM images of precipitated CaCO_3_ crystals in the presence and absence of Casein and Rhamnolipids are shown in Fig. 5. As seen, when the tested surfactants were absent, the normal cubic shape of calcite calcium carbonate crystals was detected. On the other hand, very tiny crystals, and many particles with a spherical shape similar to vaterite were observed when Rhamnolipids were present. However, all of the CaCO_3_ crystals possessed a loose framework, and their particle size was not affected after Casein’s addition. Additionally, a large number of particles transformed into unstable spherical vaterite crystals.

This modification can be explained by the presence of carboxylate and hydroxyl groups in both surfactants in addition to Casein’s amin group. Heteroatoms in these functional groups cause coordination interactions with calcium ions (Ca^2+^) in CaCO_3_ microcrystals. This interaction causes crystal growth defects leading to morphological modifications. Whereas, the presence of these functional groups and lone pair electrons on scale crystals, operate as adsorption agents to stop further crystal growth^37^.

##### X-ray diffraction (XRD)

**Fig. 6:**
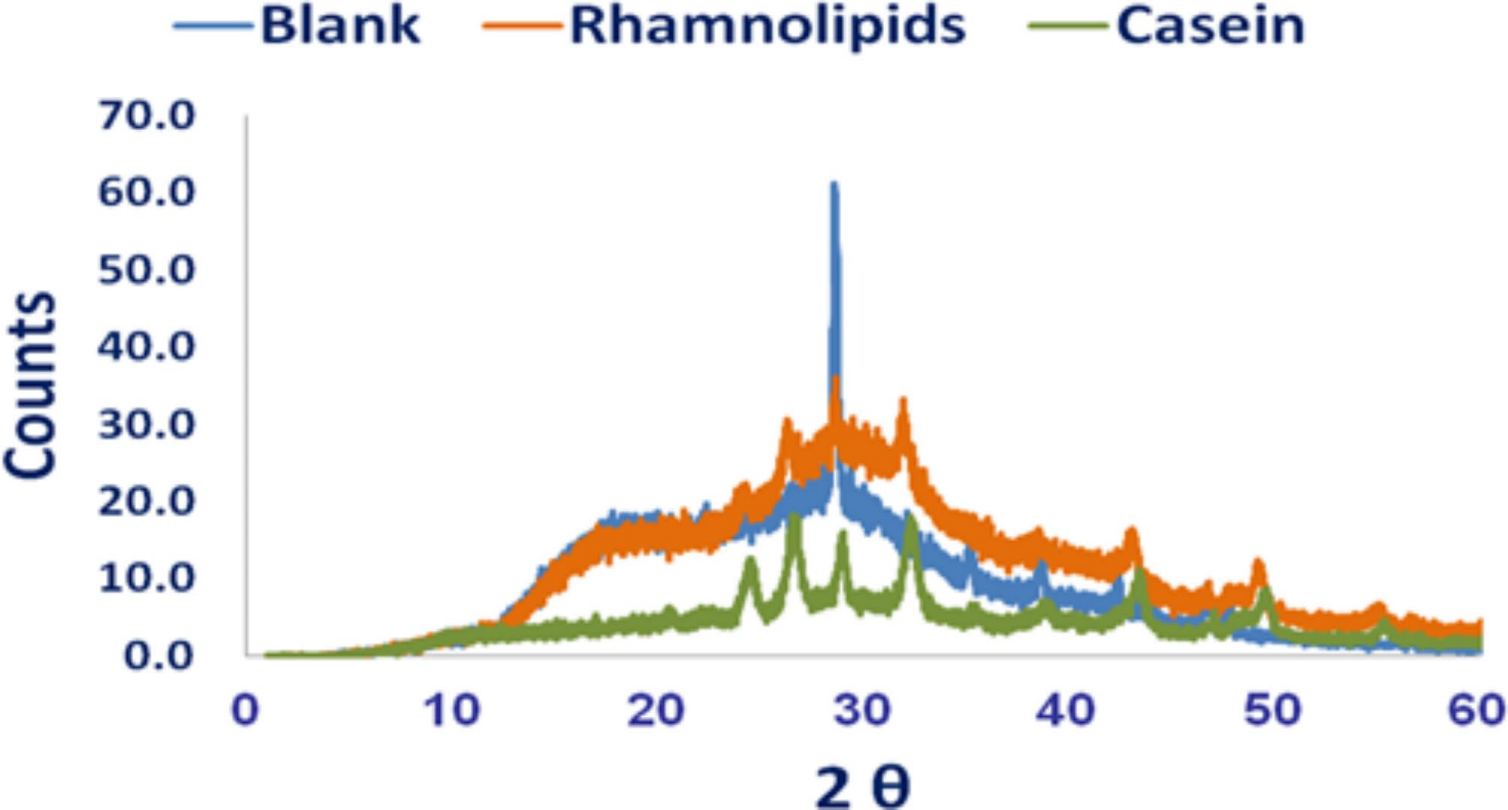
XRD results of the precipitated CaCO_3_ crystals in the absence and presence of Rhamnolipids and Casein.

For further investigation of the effect of Casein and Rhamnolipids on the crystallinity of calcium carbonate, the XRD was measured as indicated in Fig. 6. As can be seen, diffraction peaks at 2θ = 23.1°, 29.4°, 36.0°, and 39.4° correspond to calcite were identified without the scale inhibitors, demonstrating that only calcite is formed. Nevertheless, the characteristic peaks of vaterite at 24.9°, 27°, and 32.8°^38^ were found by adding Casein or Rhamnolipids, but there are clear differences in the intensity of vaterite crystals. When casein was introduced, the typical peak intensity of vaterite decreased indicating that the degree of CaCO_3_crystal modification to vaterite is lower than in the presence of Rhamnolipids, these results obtained match the SEM images.

As reported, the most thermodynamically stable crystal is calcite, while the most unstable crystal is vaterite^39^. Overall, the results showed that both scale inhibitors work together to efficiently inhibit stable scale formation and promote the conversion of CaCO_3_ nuclei to vaterite. This is most likely due to the adsorption of scale inhibitors on the crystal surface, which distort the lattice, interfere with calcite formation, and alter the CaCO_3_ scale’s structure.

### Scale inhibition mechanism of Casein and Rhamnolipids

In general, there are three basic steps in the CaCO_3_ nucleation process: (1) The induction period which is the time interval in a supersaturated solution before “measurable” crystallization begins, (2) the nucleation period characterized by the formation of microparticles after the collision of random cation-anion particles and (3) the crystal growth period to form macro crystals^38,40,41^. In a brine solution, scale inhibitors may obstruct one or more of these phases in the precipitation of scales.

**Fig. 7:**
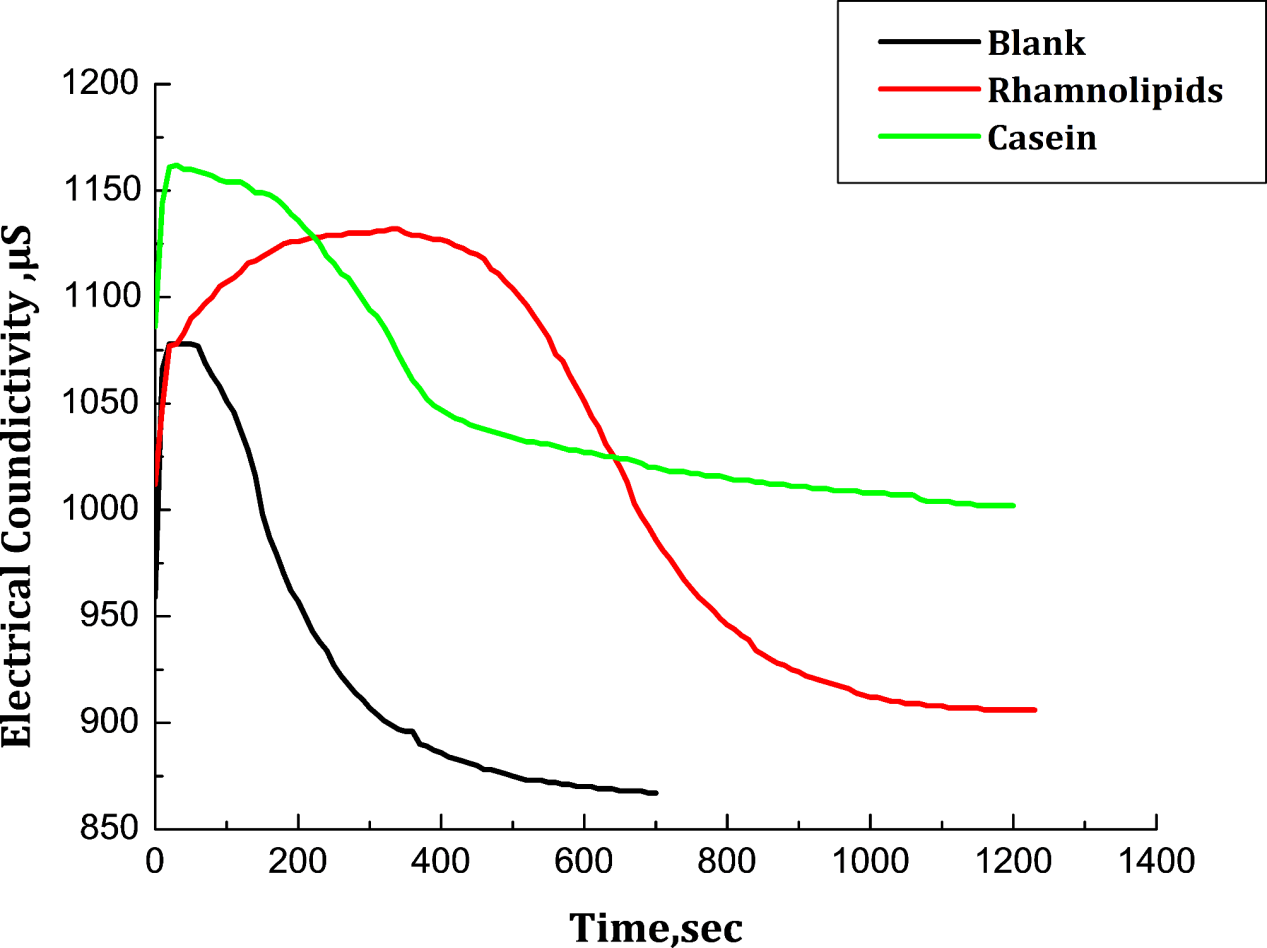
Conductivity variation with time during CaCO_3_ precipitation process.

The conductivity changes over time in the solutions containing Na_2_CO_3_ and CaCl_2_ in the presence or absence of scale inhibitors are displayed in Fig. 7. The conductivity of the crucial supersaturation CaCO_3_ solutions decreased significantly during the nucleation stage. The conductivity gradually dropped to a stable value over time; the growth and rearrangement of the CaCO_3_ nuclei is responsible for the constant conductivity value during this phase.

The observed induction time in the control experiment was 100 s, while the presence of Casein and Rhamnolipids extended the induction time to 200 and 600 s, respectively. This delay suggests that ion migration in the solution proceeds slowly. Furthermore, with the addition of Casein and Rhamnolipids, the conductivity value in the final stage of the solution is higher than that of the blank, indicating that fewer Ca^2+^ and CO_3_^2−^ ions are involved in the formation of crystal nuclei and most of the ions are free in the solution.

It was observed that the increase in nucleation and induction time follows the order:

No inhibitor < Casien < Rhamnolipids. In other words, Rhamnolipids is a much better inhibitor for calcium carbonate than Casein. This performance pattern can be explained by Casien’s tolerance to calcium. According to the literature, Casein interacts with cations (Ca^2+^) through the nitrogen atom with a high degree of tolerance^36^. Large negatively charged groups in Casein molecules can precipitate when Ca^2+^ ion is present. Casein’s high affinity for free cations in brine solution reduces the amount of scale inhibitor adsorbed on the mineral surfaces, which is an effect of its high calcium tolerance. Furthermore, tiny compounds such as Rhamnolipids diffuse much more rapidly than larger amino-based inhibitors such as casein. As a result, the nitrogen-free scale inhibitor bound to the mineral surfaces more quickly than Casien.

**Fig. 8:**
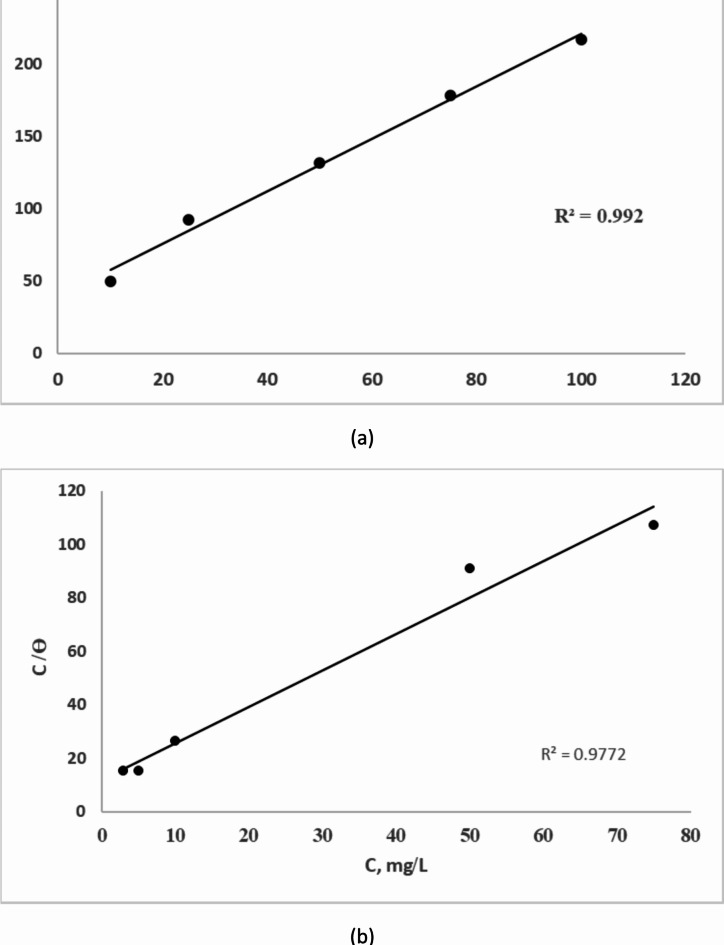
Langmuir isotherm for the adsorption of Rhamnolipids (**a**) and Casein (**b**) on calcium carbonate microcrystals.

Furthermore, the original nucleus of CaCO_3_ is more likely to be transformed into vaterite because a small number of Casien with long chains can be adsorbed on several locations of the initial nucleus. On the other hand, a significant number of short-chain Rhamnolipids can absorb into CaCO_3_ surfaces and so slow down the growth of crystals by electrostatic repulsion, resulting in the creation of smaller crystals. Figure 8 illustrates the fitting of the experimental data for the adsorption of Rhamnolipids (a) and Casein(b) on calcium carbonate microcrystals to the Langmuir isotherm which follows the equation:

2 $$\:\frac{{\text{C}}_{\text{i}\text{n}\text{h}}}{{\uptheta\:}}\:=\:\frac{1}{{\text{K}}_{\text{a}\text{d}\text{s}}}+{\text{C}}_{\text{i}\text{n}\text{h}}\:$$

Given that, C_inh_ is casein or Rhamnolipids, concentration in mg/L, K is the constant of adsorption equilibrium and θ is the coverage area^42,43^. The Langmuir model adsorption isotherm showed that scaling inhibition was caused by the adsorption of the surfactants on the crystal growth sites, which lowers the nucleation rate and prevents the formation of crystals.

The comparisons of the scale inhibition between Casein, Rhamnolipids, and some other eco-friendly scale inhibitors are listed in Table 2^24,44–50^. It is believable that these green surfactants, especially Rhamnolipids, can be considered promising green-scale inhibitors.

**Table 2:** Comparison of the scale inhibition between Rhamnolipids, Casein, and some other eco-friendly scale inhibitors.

Scale inhibitor	Scale type	Test method	Dose	Inhibition efficiency (%)	References
Rhamnolipids	CaCO_3_	Electrochemical measurements	100 ppm	81	
Casein	CaCO_3_	Electrochemical measurements	200 ppm	56	
Carboxymethyl chitosan	Ca CO_3_	Computability and dynamic tube blocking test	170 ppm	Efficient	^44^
Sodium carboxymethyl cellulose (SCMC)	CaCO_3_	pH displacement method	200 mg/L	93	^45^
Polyaspartic acid (PASP)	CaCO_3_	Standard static test	80 ppm(80 ºC)	87	^46^
			80 ppm(90 ºC)	69	
Curcumin-citric Acid-aspartic Acid Polymer (PCCA)	CaCO_3_	Standard static test	4ppm	99.7	^47^
Chitosan- alginate Mixture	CaCO_3_	Conductivity, electrochemical measurements	400:50ppm	88	^24^
MA-VA-VS (maleic anhydride, vinyl acetate (VA), vinyl sulfonate (VS) as monomers, and ammonium persulfate)	Ca CO_3_	Static jar measurement and dynamic measurement	150 mg/L	91.4	^48^
Chitosan derivative (O-CTS-PSI)	Ca CO_3_	Standard static test	40 mg/L	100	^49^
Poly (aspartic acid)-modified polymer	CaCO_3_	Standard static test and electrochemical methods	40 mg/L	65.32	^50^

### Computational study

The optimal approach for discerning the distinct impacts of two green surfactant compounds lies in utilizing the DFT (Density Functional Theory) calculation method. This technique relies on information derived from the spatial and electronic configuration of an optimized molecular structure. The optimized geometry of both Casein and Rhamnolipids is illustrated in Supplementary Fig. S3, providing a visual representation of their molecular arrangements, and the parameters is shown in Table S1. Table 3, presents the quantum chemical parameters obtained through calculations performed at the B3LYP/6–31 g(d, p) level of theory for casein and rhamnolipids molecules. In the realm of quantum chemistry, certain parameters hold significance in understanding the behavior of molecules. Notably, high values of E_(HOMO )_are correlated with enhanced adsorption on a metal surface and improved inhibition efficiency, as indicated by references^51,52^. Conversely, a lower energy level of the E_(LUMO )_orbital suggests a higher likelihood of the molecule accepting electrons from superficial metallic atoms, as supported by references^53,54^. Defined as the difference between LUMO and HOMO energies, the energy gap (∆E_gap_) is an important measure that indicates a molecule’s kinetic stability and chemical reactivity. Low chemical reactivity and good kinetic stability are indicated by a big energy gap. Conversely, as references confirm, low values show substantial interactions between reacting species and are linked to better inhibitory qualities^55–57^. Analyzing the data presented in Table 3, it becomes evident that rhamnolipids exhibit superior inhibitory properties compared to casein for industrial applications.

**Table 3:** Quantum parameters for Casein and Rhamnolipids inhibitor.

	Casien	Rhamnolipids
$\:{E}_{HOMO}\left(eV\right)$	− 5.22	− 4.61
$\:{E}_{LUMO}\left(eV\right)$	− 0.50	− 1.21
$\:{\Delta\:}{E}_{gap}\:\left(eV\right)$	4.72	3.40
$\:\eta\:\:\left(eV\right)$	2.36	1.70
$\:\sigma\:\:{\left(eV\right)}^{-1}$	0.424	0.588
$\:\mu\:\:\left(eV\right)$	− 2.86	− 2.91
$\:\chi\:\left(eV\right)$	2.86	2.91
$\:\omega\:\:\left(eV\right)$	1.73	2.49
$\:{\omega\:}^{+}\left(eV\right)$	0.59	1.25
$\:{\omega\:}^{-}\left(eV\right)$	3.46	4.16
$\:{{\Delta\:}E}_{\text{b}\text{a}\text{c}\text{k}-\text{d}\text{o}\text{n}\text{a}\text{t}\text{i}\text{o}\text{n}}\left(eV\right)$	− 0.59	− 0.43
Q_NBO_(e)	(O atoms − 0.57 to − 0.72) (N atoms − 0.63 to − 0.91)	(O atoms − 0.44 to − 0.73)

**Fig. 9:**
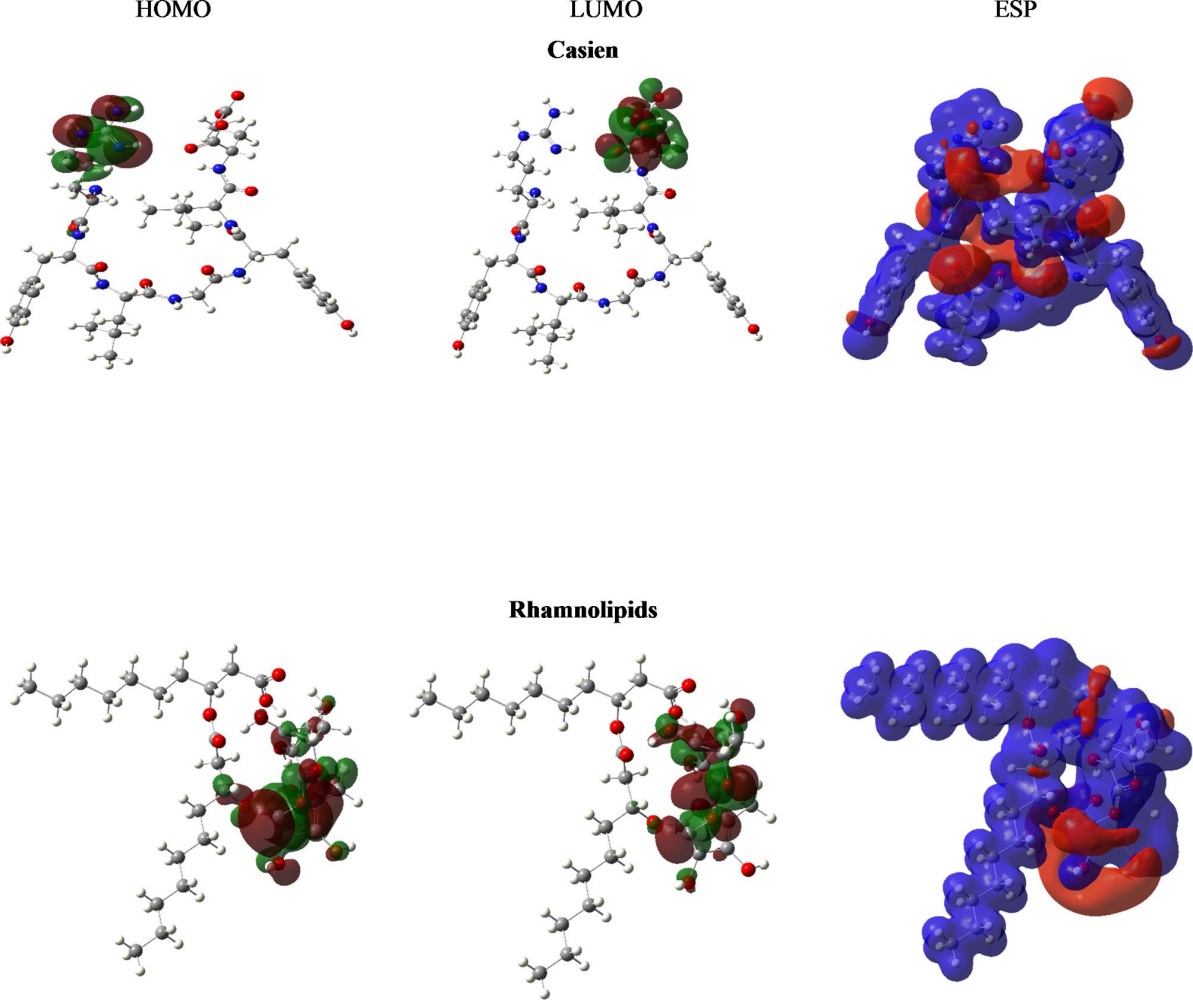
Frontier molecular orbitals and electrostatic potential maps for Casein and Rhamnolipids.

**Fig. 10:**
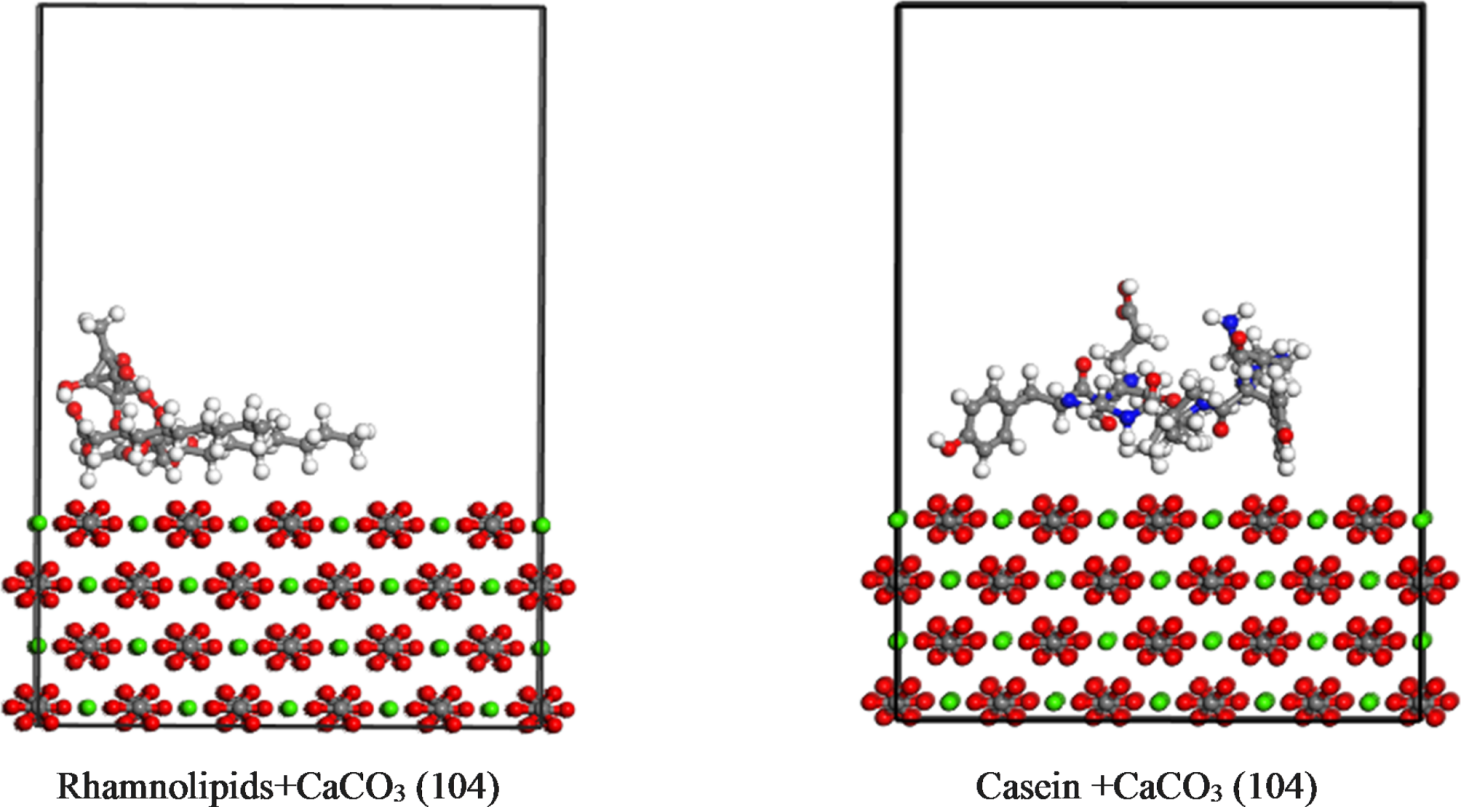
Adsorption geometry of Rhamnolipids and Casein on CaCO_3_ (104) surface.

This study examined the local reactivity of inhibitor compounds and determined their preferred adsorption sites on crystal surfaces using frontier molecular orbital (FMO) plots and electrostatic potential (ESP) maps (Fig. 9). Figure 10 illustrates that in the HOMO of casein, the electron density is dispersed across the guanidine moiety, while in the LUMO, the electron density is spread over the glutaric acid. Conversely, for Rhamnolipids, both the HOMO and LUMO surfaces are distributed over the rhamnose moiety. This observation implies that the molecule possesses the capacity to both donate and accept electrons, suggesting the involvement of donor–acceptor interactions in the adsorption of this molecule onto the adsorbent surface^58^.

Upon examining the inhibitor molecules’ electrostatic potential (ESP) mappings, we found that the nitrogen atoms of rhamnolipids and the oxygen and nitrogen atoms of casein were the primary locations of notable negative potential areas, denoted by red regions in Fig. 9. On the other hand, the inhibitor molecule’s remaining skeleton was characterized by blue areas that indicated positive potential. The data mentioned above indicate that the spots with a higher electron density and negative potential are more favorable for adsorption on crystal surfaces, possibly due to their capacity to interact positively with Ca^2+^ ions.

To assess a molecule’s stability and reactivity, its softness (σ) and absolute hardness (η) serve as well-established characteristics. Chemical hardness denotes the resistance to displacement or polarization of electron clouds within molecules, ions, and atoms when subjected to minor perturbations during chemical reactions. A molecule is considered soft when its energy gap is small, while it is deemed hard when the energy gap is substantial. The adsorption of an inhibitor onto a metal surface occurs in the softest and least hardened region of the molecule. Computed values for the selected molecules as inhibitors are presented in Table 3. A comparison between the two inhibitors reveals that the Casein inhibitor possesses the highest hardness value. This trend suggests that a lower hardness value, as observed in the Rhamnolipids inhibitor (and consequently the highest global softness value), may result in best inhibitory effectiveness.

Chemical potential (µ), electronegativity (χ), and the global electrophilicity index (ω) are essential indicators for assessing the chemical reactivity of molecules concerning their adsorption on surfaces^59^. The chemical potential, which signifies the change in total electronic energy relative to the number of electrons, is essentially the negative counterpart of electronegativity. The global electrophilicity index (ω) is expressed in relation to the global hardness and electronegativity parameters. Elevated values of χ and ω suggest a molecule’s electrophilic nature, indicating its capacity to accept electrons and making it more reactive towards electron-rich species (nucleophiles). Lower values of χ and ω imply an electron-donating capacity and a nucleophilic character^60^. Table 3 summarizes the values of the current system. A comparison reveals that Rhamnolipids exhibit higher electronegativity and electrophilicity power values compared to Casein. This indicates that Rhamnolipids serve as a better scale inhibitor than the casein molecule. As seen in Table 3, Rhamnolipid shows a higher electron-accepting power compared to Casein, suggesting it can more readily accept electrons in interactions with the surface or other species. Both inhibitors exhibit a strong electron-donating ability, with Rhamnolipid having a slightly higher value, indicating it can also donate electrons more effectively, which might influence its adsorption characteristics on CaCO₃ (104) surfaces. Back-donation energy (ΔE_back−donation_) values show that both inhibitors can participate in electron back-donation with the surface, contributing to their protective capabilities.

The NBO charges highlight the key role of oxygen atoms in Rhamnolipids and the combined role of oxygen and nitrogen atoms in Casein as seen in Table 3. While Rhamnolipids have slightly stronger adsorption energy, the nitrogen atoms in Casein could provide more versatile interactions, making it a competitive alternative. The difference in adsorption energies suggests that both inhibitors are effective, with Rhamnolipids potentially offering a slightly stronger interaction, while Casein could benefit from its additional nitrogen-based adsorption sites.

The assessment of inhibition efficiency by rhamnolipids and casein involves their adsorption on the CaCO_3_ (104) surface, recognized as the most stable plane for calcite in neutral and slightly basic conditions. Monte Carlo simulations were employed to analyze the adsorption characteristics, enabling the calculation of stable adsorption configurations and their corresponding adsorption energies. The adsorption energy (Eads) was determined using the formula:

3 $$\:{E}_{ads}={E}_{complex}-{E}_{\text{A}\text{d}\text{s}\text{o}\text{r}\text{b}\text{a}\text{t}\text{e}}-{E}_{\text{S}\text{u}\text{b}\text{s}\text{t}\text{r}\text{a}\text{t}\text{e}}$$

where E_complex_ refers to the energy of the substrate-adsorbate configuration, E_Adsorbate_, and E_Substrate_ are the energy of the scale inhibitor molecule and the metal surface, respectively.

The low-energy equilibrium configurations of the rhamnolipids and casein structures adsorbed on CaCO_3_ (104), as obtained through Monte Carlo simulations, are illustrated in Fig. 10. The orientation of all inhibitor molecules parallel to the CaCO_3_ surface is evident as a consequence of adsorption. The resulting adsorption energies, detailed in Table 4, reveal values of − 23.84 kcal/mol and − 21.75 kcal/mol for Rhamnolipids and Casein molecules, respectively. The negative sign in the adsorption energies indicates a favorable adsorption process, with lower values suggesting stronger interactions. In this context, the obtained adsorption energies of − 23.84 kcal/mol for rhamnolipids and − 21.75 kcal/mol for casein imply that rhamnolipids exhibit a more effective inhibition compared to casein. This is attributed to the higher magnitude of the adsorption energy for Rhamnolipids, signifying a more stable and stronger binding to the CaCO_3_ (104) surface. To provide a comprehensive assessment of the adsorption performance of Rhamnolipids and Casein relative to established inhibitors such as polyacrylic acid (PAA) and salicylhydroxamic acid (SHA), a comparison of their adsorption energies can be seen in Table 4^61,62^, which provide adsorption of polyacrylic acid (PAA) and salicylhydroxamic acid (SHA), Rhamnolipids and Casein show moderate adsorption energies, which suggest that they are effective inhibitors, with adsorption strength stronger than SHA but weaker than PAA. Their moderate energies reflect a balance between stability and reactivity, making them suitable for green-scale inhibitors with promising adsorption capabilities, offering an eco-friendly alternative to traditional inhibitors of CaCO_3_ precipitation.

**Table 4:** Comparison of the adsorption energy (Kcal/mol) between Rhamnolipids, Casein, and some other scale inhibitors.

Scale inhibitor	Scale type	Test method	Adsorption energy (Kcal/mol)	References
Rhamnolipids	CaCO₃ (104)	DFT	− 23.84	
Casein	CaCO₃ (104)	DFT	− 21.75	
Polyacrylic acid (PAA)	CaCO₃ (104)	DFT	− 28.23	^61^
Salicylhydroxamic acid (SHA)	CaCO₃ (104)	DFT	− 14.15	^62^

**Fig. 11:**
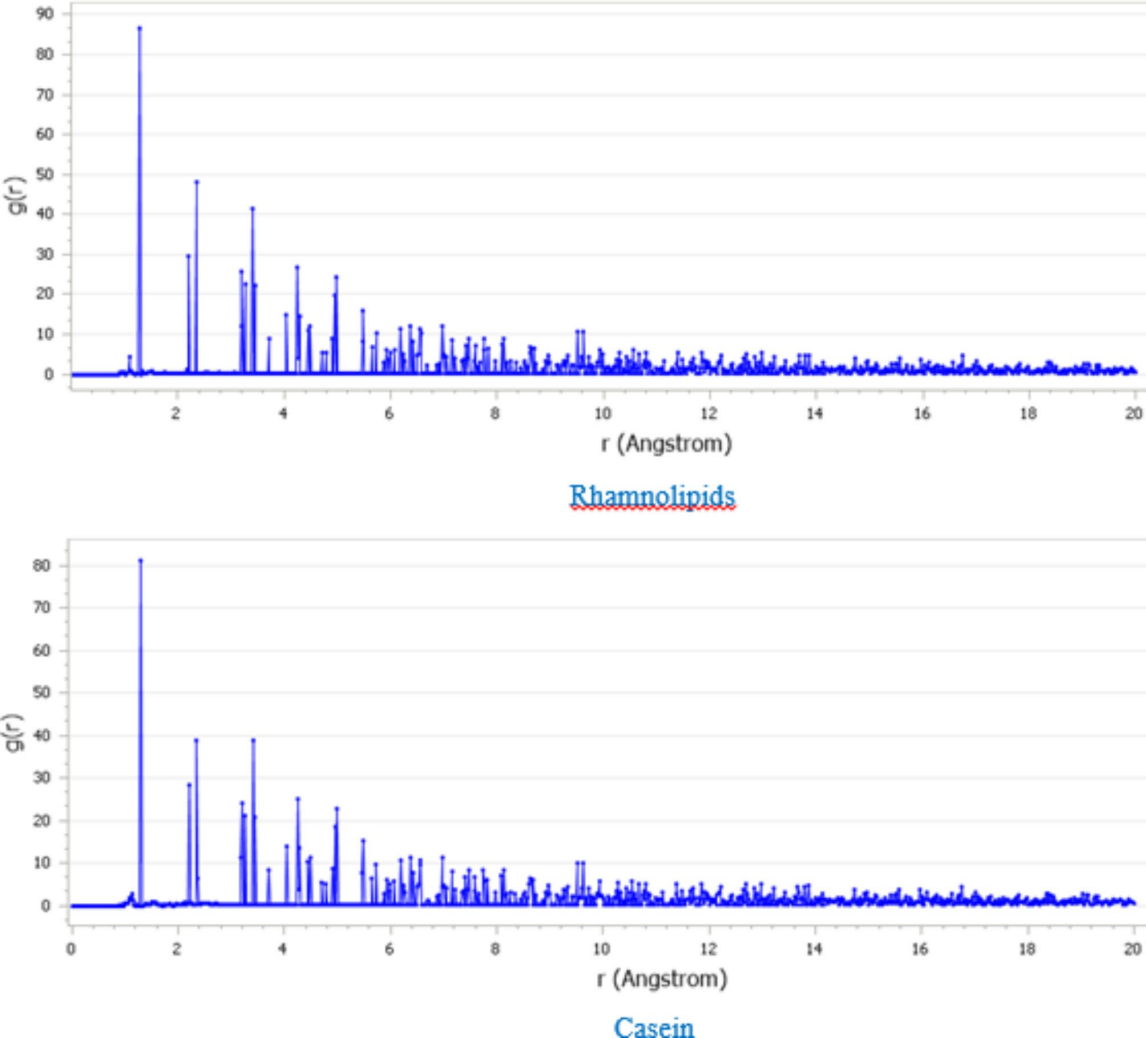
RDF analysis of Rhamnolipids and Casein on CaCO_3_ (104) surface.

As seen in Fig. 11, the Radial Distribution Function (RDF) analysis is used to assess the bond lengths between the Casein and Rhamnolipid inhibitors and the CaCO_2_ (104) surface, providing a quantitative understanding of the adsorption mechanism. Bond lengths play a pivotal role in distinguishing between chemisorption and physisorption. The bond lengths between the inhibitors (Casein and Rhamnolipid) and the CaCO_2_ (104) surface fall within the range of 1 Å to 3.5 Å, indicating that the adsorption mechanism is chemisorption. The moderate adsorption capability of Casein and Rhamnolipid, as evidenced by these bond lengths, suggests that their interaction with the CaCO_2_ surface is not as strong as traditional chemisorption. This intermediate behavior is likely a result of the specific molecular structures of these green inhibitors, which allow them to interact with the surface via both chemisorptive and physisorptive forces, leading to stable but moderate adsorption. Thus, these findings affirm that Casein and Rhamnolipid possess significant adsorption potential, particularly in the context of preventing CaCO_2_ scale formation.

## Materials and methods

### Solutions preparation

The solutions were prepared using double-distilled water, analytical reagent-grade NaCl, NaHCO_3_, Na_2_SO_4_, and CaCl_2_ (from ISO-CHEM CO., France, and Al-Gomhoria CO., Egypt). Rhamnolipid and Casein were obtained from AGAE Technologies, USA, and Alpha chemika, India, respectively. Casein was dissolved in sodium hydroxide to form a stock solution, and the pH was then adjusted to 8.18.

### Conductivity test for CaCO_3_ scaling

According to earlier instructions, a conductivity test was carried out to test the recommended inhibitor^34^. In each experiment, the conductivity of the stirred solution was monitored during titration with 0.01 M Na_2_CO_3_ using HQ14d conductivity meter. The titrant was added in 0.2 mL increments. The test identifies the point of supersaturation at which precipitation of CaCO_3_ begins which is accompanied by a notable decrease in solution conductivity. The measurements were performed at 25.0 ± 0.1 °C.

### Examination for CaCO_3_ scaling electrochemically

This test used a three-electrode mode cell, with platinum wire (counter electrode) and a saturated calomel electrode (SCE) (reference electrode) for the electrochemical experiments in synthetic cooling water^63^. The working electrode steel’s chemical composition was as follows (wt%): Mn = 2.5, *P* = 0.04, Si = 0.35, C = 0.21, S = 0.04, and Fe in balance. To precipitate CaCO_3_ scales and measure electrochemical impedance, the Gamery instrument G300TM Potentiostat/Galvanostat/ZRA was utilized. To accelerate calcium carbonate precipitation, using the following equation, the steel electrode was polarized for three hours to − 0.95 V (vs. SCE) in the test solution:

4 $${\text{C}}{{\text{a}}^{{\text{2}}+}}+{\text{ HC}}{{\text{O}}_{\text{3}}}^{ - }+{\text{ O}}{{\text{H}}^ - }~{\text{CaC}}{{\text{O}}_{\text{3}}}\,+\,{{\text{H}}_{\text{2}}}{\text{O}}$$

Following the scale deposition process using the previous cathodic polarization, EIS measurements were carried out. EIS measurements were conducted between 0.1 and 1 × 104 Hz, with an applied potential signal amplitude of 10 mV. Every measurement was conducted at 40.0 ± 0.1 °C. In every instance, experiments were conducted in triplicate under identical conditions to assess the consistency and reliability of the experiments.

### Morphological examination

#### SEM

Using a scanning electron microscope (JEOL-5300), the morphology and crystal lattices of the scale were examined. The scale crystals were subjected to a thin layer of gold vacuum sputter coating before SEM examination.

### X-ray diffraction

An X-ray powder diffractometer-XRD-D2 phaser, Bruker, Germany, was used for the X-ray powder diffraction investigation. Cu Ka radiation (40 kV and 30 mA), and a Ni filter were used with a scanning speed of 0.005° 2q s21.

### Computational details

The DFT approach used in this study combines the gradient-corrected correlation functional of Lee, Yang, and Parr (LYP) with Hartree-Fock (HF) with DFT exchange terms, incorporating Becke’s three-parameter functional (B_3_)^64^. In this calculations, 6–31 g(d, p) basis set were used. Computational chemistry has been a great benefit in the design and development of chemical inhibitors, where DFT playing a crucial role^65^. Gaussian 09 software was employed for DFT calculations, encompassing geometry optimization for both rhamnolipids and casein compounds^66^. Theoretical frameworks such as DFT make it easier to quantitatively calculate global indices that characterize the intrinsic reactivity of chemical species. Interestingly, our calculations yielded a number of reactivity descriptors such as electron affinity (A), ionization potential (I), the gap (ΔE_gap_), electronegativity (χ), chemical potential (µ), electrophilicity (ω), global hardness (η), electron-accepting power (ω⁺), electron-donating power (ω⁻), and back-donation (ΔE_back−donation_)^67–69^.

5 $$\:\varDelta\:{\varvec{E}}_{\varvec{g}\varvec{a}\varvec{p}}=\:{\varvec{E}}_{\varvec{L}\varvec{U}\varvec{M}\varvec{O}}-{\varvec{E}}_{\varvec{H}\varvec{O}\varvec{M}\varvec{O}}$$

6 $$\:\eta\:=\frac{{\Delta\:}{E}_{gap}}{2},\sigma\:=\frac{1}{\eta\:}$$

7 $$\:\chi\:=-\frac{{(E}_{HOMO}+{E}_{LUMO})}{2}$$

8 $$\:\mu\:=\:\frac{{(E}_{HOMO}+{E}_{LUMO})}{2}$$

9 $$\:\omega\:=\:\frac{{\mu\:}^{2}}{2\eta\:}\:$$

### Monte Carlo and molecular dynamics simulation

The simulation of adsorption and binding interactions between the rhamnolipids and casein inhibitor and the calcite surface was accomplished through the implementation of the Monte Carlo simulation technique. This involved using Accelrys’ Materials Studio 2016 software’s adsorption locator module^70^. Through the simulation of a single inhibitor molecule’s adsorption on the surface of the CaCO_3_(104) crystal, the interaction between the compounds under investigation and the surface was modeled. The CaCO_3_(104) surface was split into a 104 plane to create a CaCO_3_ (104) crystal plane, which was then expanded to form a 5 × 5 supercell. Additionally, a void slab measuring 25Å was constructed on top of the plane. To determine the adsorption properties of rhamnolipids and casein, simulated annealing calculation was employed, utilizing the COMPASS II force field and the Ewald summation method. This approach ensured fine-quality simulation results, allowing for a comprehensive exploration of the adsorption behavior and binding interactions of the scale inhibitors with the calcite surface. The use of Monte Carlo simulations, coupled with advanced modeling techniques, not only facilitated a detailed investigation into the molecular-level interactions but also provided a thorough understanding of the adsorption processes occurring at the CaCO_3_(104) surface. Also, molecular dynamics simulations were performed using the NVT ensemble, employing the Andersen thermostat with a time step of 1.0 femtoseconds, over duration of 20 picoseconds at a temperature of 298 K, with the COMPASSII force field utilized for interaction modeling.

## Conclusions

This extensive study, utilizing advanced experimental and computational approaches, offers valuable insights into the potential of Rhamnolipids and Casein as eco-friendly scale inhibitors for water treatment. Experimental findings indicate that Rhamnolipids exhibit superior anti-scaling performance compared to casein, despite Casein’s higher content of functional groups. Morphological analyses demonstrate that both inhibitors effectively prevent the formation of stable calcite crystals and promote the transformation of CaCO_3_ nuclei into unstable vaterite. This behavior likely results from the adsorption of inhibitor molecules on the crystal surface, leading to lattice distortion that impacts calcite formation.

Integrating DFT calculations, molecular orbital analysis, and Monte Carlo simulations allows a comprehensive exploration of the molecular-level interactions, reactivity descriptors, and adsorption characteristics of these eco-friendly surfactant molecules on the CaCO_3_ (104) surface. The obtained adsorption energies of − 23.84 kcal/mol for rhamnolipids and − 21.75 kcal/mol for casein imply that rhamnolipids exhibit a more effective inhibition compared to casein. The results consistently highlight Rhamnolipids as the most effective inhibitor, attributed to its distinctive electronic structure, and more favorable adsorption energies. Overall, the results revealed that naturally based surfactants exhibit good performance in inhibiting calcium carbonate and it can serve as a viable alternative to conventional, non-green water treatment technologies.

### Future scope

Building on the findings of this study, naturally based surfactants show significant promise as inhibitors for calcium carbonate scales, a prevalent issue in the industrial sector. However, it is essential to address the limitations of these green materials. Future research should focus on assessing the stability and biodegradability of bio-based surfactants to enhance their shelf life and practical applicability. Additionally, scaling-up efforts should include evaluating the performance of these materials under varying industrial conditions, such as different temperatures, pressures, and flow rates, to ensure their effectiveness in real applications.

## Electronic supplementary material

Below is the link to the electronic supplementary material.

Supplementary Material 1

## Data Availability

All data generated or analyzed are included within this article or in supplementary information.
