# Supplementary material for: Innovative application of green surfactants as eco-friendly scale inhibitors in industrial water systems
Source: Sci Rep. 2024 Nov 14;14:28073. doi: 10.1038/s41598-024-78879-1 (PMC11564881; doi:10.1038/s41598-024-78879-1)
Supplement: Supplementary file 1 — Supplementary Material 1 [file 41598_2024_78879_MOESM1_ESM.docx]

**Supplementary Information**

Table S1: optimized geometrical parameters for Rhamnolipid and Casein molecules.

|  | Rhamnolipid | Casein |
| --- | --- | --- |
| Bond distance, Å | 1.26 (O1-C2)  1.47 (C2-C3)  1.53 (C3-C4)  1.46(C4-C5)  1.49(C5-C6)(C1-C15)(C15-C16)  1.48(C6-O8)  1.35 (C4-O11)(C3-O13)(C5-O9)(C20-O22)  1.54(C16-C47)  1.45(C16-O43)(C23-O22)  1.41(C44-O43)  1.34(C44-C46)  1.36(C46-O50)  1.38(C16-O17)  1.54(C18-C19)  1.22(C20-O21)(C25-O26)  1.53(C29-C30) | 1.54(C1-C2)(C4-C5)  1.22(C6-O7)(C10-O11)(C22-O23)  (C30-O31)(C39-O40)  1.36(C6-N8)(C10-N12)(C30-N32)  1.45(C45-N54)(C57-N65)  1.51(C43-C45)  1.35(C36-O38) |
| Angle (°) | 118.4(O1-C2-O8)  88.8(C3-C4-C6)  127.6(C2-O1-C15)  62.9(C47-C15-C16)  106.3(C47-C16-O43)  117.7(C16-O17-C18)  108.2(O17-C18-C36)  106.9(O22-C23-C24) | 119.8(C5-C6-N8)  119.6(C9-C10-O11)  111.2(N12-C13-C14)  119.1(C20-C15-C16)  123.0(C22- N24-C25)  110.3 (N32- C33-C34)  120.9(C35-C36-O38)  118.9(O40-C39-O41) |

| 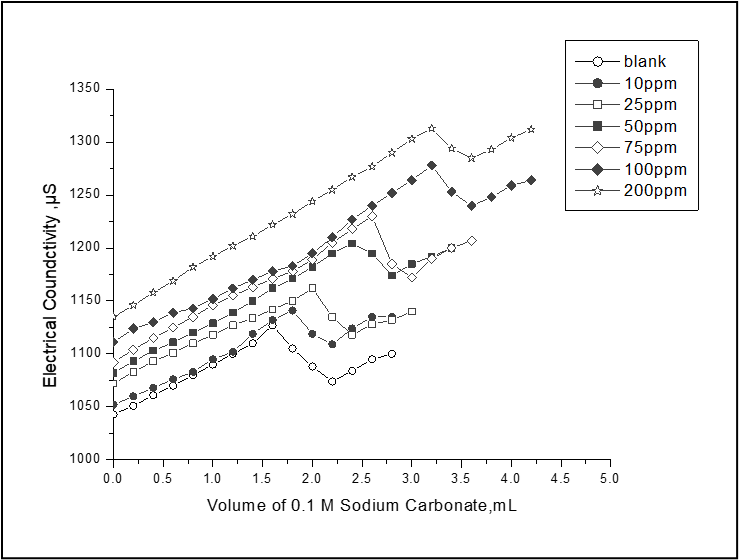 |
| --- |
| **a** |
| 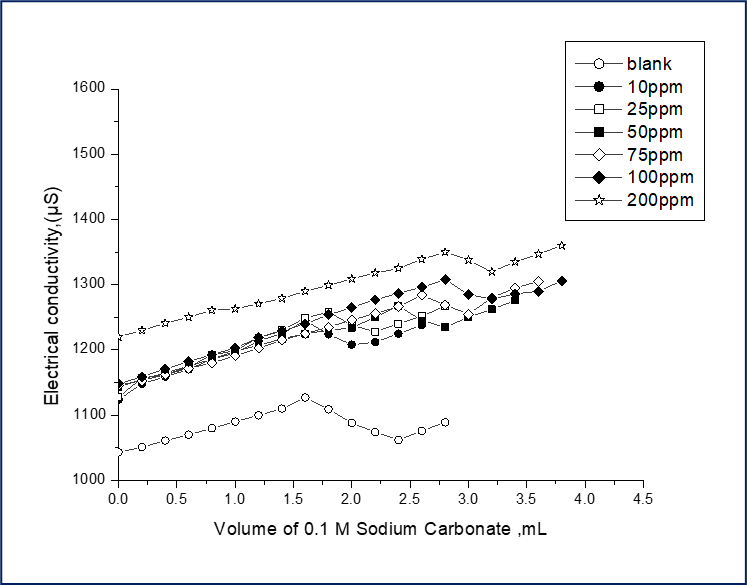 |
| **b** |

**Figure S1:** Conductivity variation of 0.1 M CaCl_2_ solution with volume of added Na_2_CO_3_ without and with different Rhamnolipids (a) and Casein (b) concentrations.

**
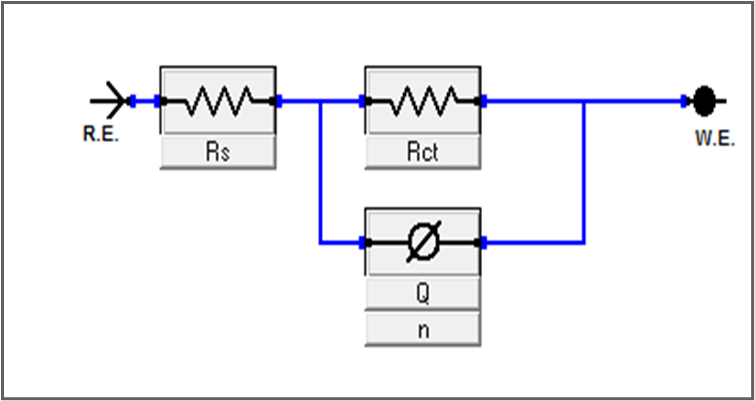
**

**Figure S2:** The equivalent circuit model's characteristics of impedance that were used to calculate the scaling parameters in the CaCl_2_ brine solution both with and without scale inhibitors.

**(R_s_: the solution resistance, R_ct_: the charge transfer resistance, Q is associated to the double-layer capacitance)**

|  |
| --- |
|  |
| Rhamnolipids |
| **** |
| Casein |

**Figure S3:**Impedance spectra of polarized steel in brine solution with its fitting curves and equivalent circuits in the absence and presence of scale inhibitors.

| Rhamnolipid | 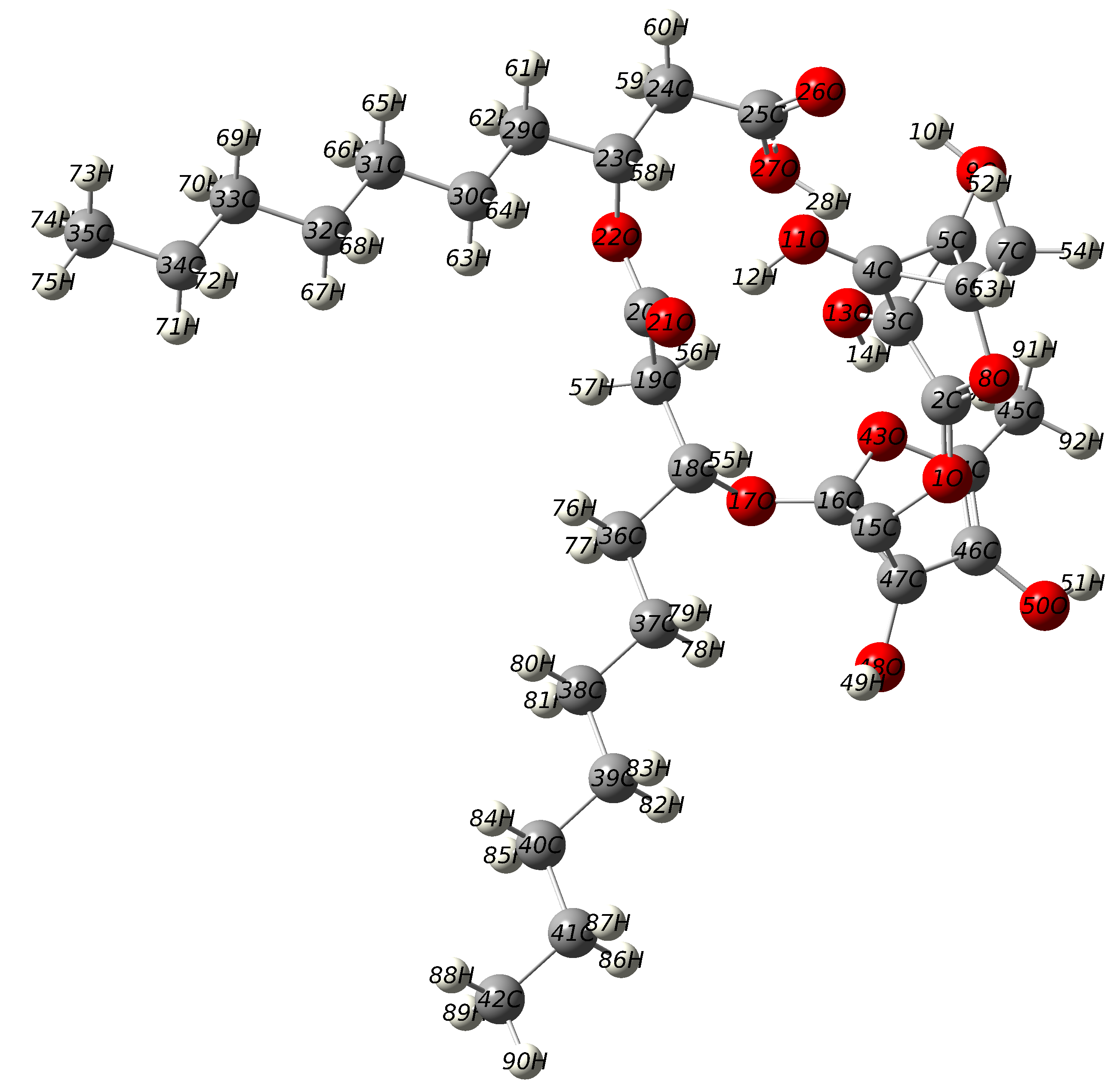 |
| --- | --- |

| Casein | 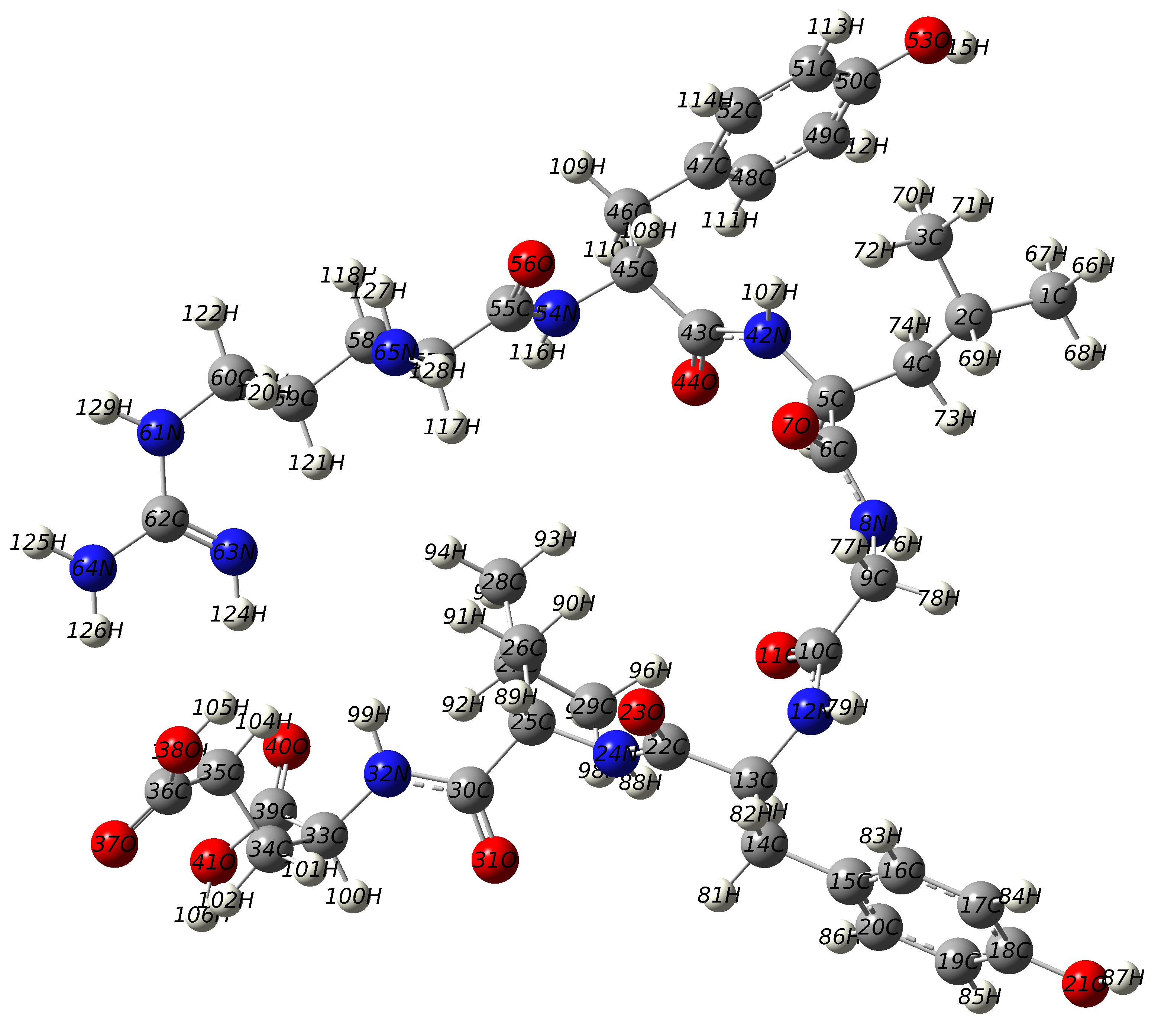 |
| --- | --- |

Fig. S4: Optimized geometries of Rhamnolipid and Casein molecules.
